# Gut Microbiota–Metabolite Alterations Associated with Early Metabolic Dysfunction and Hepatic Steatosis in Adolescents

**DOI:** 10.34133/csbj.0136

**Published:** 2026-08-11

**Authors:** Natalia Zeber-Lubecka, Paweł Czarnowski, Joanna Ziemska-Legięcka, Aldona Wierzbicka-Rucińska, Wojciech Jańczyk, Jacek Michałkiewicz, Łukasz Obrycki, Mieczysław Litwin, Michał Mikula, Piotr Socha, Jerzy Ostrowski

**Affiliations:** ^1^ Department of Gastroenterology, Hepatology and Clinical Oncology, Centre of Postgraduate Medical Education, 02-781 Warsaw, Poland.; ^2^ Department of Experimental Oncology, Laboratory of Cancer Metabolism, Maria Sklodowska-Curie National Research Institute of Oncology, 02-781 Warsaw, Poland.; ^3^ Department of Clinical Biochemistry, The Children’s Memorial Health Institute, 04-730 Warsaw, Poland.; ^4^ Department of Gastroenterology, Hepatology and Nutrition Disorders, The Children’s Memorial Health Institute, 04-730 Warsaw, Poland.; ^5^ Department of Microbiology and Clinical Immunology, The Children’s Memorial Health Institute, 04-730 Warsaw, Poland.; ^6^ Department of Nephrology, Kidney Transplantation and Hypertension, The Children’s Memorial Health Institute, 04-730 Warsaw, Poland.

## Abstract

**Background:** Overweight, obesity, and metabolic dysfunction-associated fatty liver disease (MASLD) are increasingly prevalent in adolescents and are linked to alterations in the gut–liver axis. Gut microbiota may contribute to early metabolic disturbances preceding overt disease. **Objective:** To compare gut microbiota composition and fecal metabolite profiles, including short-chain fatty acids (SCFAs) and amino acids (AAs), between adolescents with overweight/obesity and normal-weight peers, and to evaluate differences associated with hepatic steatosis assessed by FibroScan-controlled attenuation parameter (CAP). Of 111 participants aged 13 to 17 years, 83 and 28 represented the study group with overweight or obesity and normal-weight control group, respectively. Hepatic steatosis consistent with MASLD was defined as CAP ≥250 dB/m. Gut microbiota was assessed by 16S ribosomal RNA (rRNA) sequencing and fecal metabolites by gas chromatography–mass spectrometry (GC-MS). **Results:** Adolescents with overweight/obesity showed impaired metabolic profiles compared with controls, while overall microbial α and β diversity did not differ between groups. Differences were observed in the relative abundance of several bacterial genera, including depletion of *Lactobacillus*. Fecal concentrations of most AAs were significantly elevated in the overweight/obesity group, whereas SCFA levels were unchanged. CAP-based stratification revealed that hepatic steatosis was associated with differences in richness-related α-diversity indices without β-diversity separation. Adolescents with fatty liver exhibited a distinct microbial signature involving low-abundance taxa and higher fecal acetate and butyrate levels. **Conclusions:** Adolescents with overweight/obesity and fatty liver display subtle but biologically relevant alterations in gut microbiota composition and fecal metabolite profiles despite preserved global microbial diversity. Early metabolic impairment appears to be associated with changes in specific bacterial taxa and AA metabolism rather than generalized dysbiosis.

## Introduction

Between 2015 and 2017, nearly 30% of children across 36 European countries were overweight and 10% had obesity [1,2]. Obesity in both children and adults is the leading cause of chronic fatty liver disease, traditionally known as nonalcoholic fatty liver disease (NAFLD). More recently, NAFLD has been replaced by the proposed terms metabolic dysfunction-associated fatty liver disease (MASLD) and metabolic-associated fatty liver disease (MAFLD). These definitions not only exclude alcohol as an etiological factor but also emphasize that metabolic disturbances such as obesity, diabetes, and dyslipidemia are the main drivers of fatty liver disease [1,3–5]. The global prevalence of MASLD in pediatric populations ranges from 7% to 35%, depending on diagnostic methods, population characteristics, and geographical region [6].

Regardless of the nomenclature, fatty liver is related to genetic, hormonal, and lifestyle-associated factors [7–10] and is closely associated with comorbidities such as hypertension and type 2 diabetes [11,12]. It is also accompanied by chronic low-grade inflammation in adipose tissue, skeletal muscle, liver, pancreatic islets, intestine, and brain. Since lifestyle and dietary patterns are considered responsible for changes in gut microbiota composition and function, the bidirectional relationship between the gut microbiota and its metabolites and the liver (the gut–liver axis) may play a fundamental role in the pathogenesis of MASLD [13,14]. The development of pediatric MASLD may also depend on altered microbiota maturation and early-life exposures, including antibiotics [15]. Although findings across different studies have been inconsistent, a meta-analysis of 15 studies demonstrated a distinct gut microbial profile in patients with MASLD with higher abundance of *Escherichia, Prevotella*, and *Streptococcus* and lower abundances of *Coprococcus*, *Faecalibacterium*, and *Ruminococcus* [16]. In nonobese MASLD, higher abundances of *Ruminococcaceae* and *Veillonellaceae* were associated with hepatic fibrosis severity [17]. Reduced microbial diversity, together with a decrease in anti-inflammatory microbes and an increase in pro-inflammatory microbes, was also reported [18]. However, the molecular mechanisms that drive the initiation and progression of metabolic dysfunction still remain under investigation [13].

While liver biopsy remains the gold standard for diagnosing and staging fatty liver disease, a CAP score generated by FibroScan is an accepted noninvasive ultrasound-based tool that quantifies steatosis of the liver [19,20]. The aim of this study was to compare gut microbial parameters, determined by fecal 16S ribosomal RNA (rRNA) sequencing and mass spectrometric analyses of selected short-chain fatty acids (SCFAs) and amino acids (AAs), between adolescents with overweight/obesity compared with normal-weight controls, and then between subgroups of individuals with overweight/obesity divided according the CAP score for those with and without fatty accumulation in the liver.

## Materials and Methods

### Study design and ethic

This was a cross-sectional observational study conducted at the Children’s Memorial Health Institute in Warsaw, Poland. The study was approved by the Ethics Committee of the Children’s Memorial Health Institute (approval no. 34/KBE/2019). A detailed explanation of the study was provided to all participants and their parents. Written informed consent was obtained from all parents and from participants prior to enrollment. All procedures adhered to institutional and national ethical guidelines and complied with the principles of the Declaration of Helsinki and its subsequent amendments.

### Study population

A total of 111 adolescents aged 13 to 17 years were recruited at the Children’s Memorial Health Institute in Warsaw, Poland. Among them, 83 participants with overweight or obesity constituted the study group, and 28 normal-weight adolescents served as the control group. Overweight and obesity were defined according to the International Obesity Task Force (IOTF) criteria. The inclusion criterion was the informed consent of the participants and their parents to participate in the study. Exclusion criteria included (a) autoimmune diseases, chronic inflammatory conditions, or genetic syndromes associated with obesity; (b) use of antibiotics, probiotics, prebiotics, or synbiotics within 3 months prior to fecal sample collection; (c) use of medications affecting lipid metabolism or liver function; (d) psychiatric illness; and (e) incomplete clinical data or lack of consent to participate in the study.

### Sample collection

Fecal samples were collected from all participants at the Children’s Memorial Health Institute using a dedicated collection kit containing a sterile tube with a spatula, a Styrofoam box, and an ice pack. Samples were immediately stored at −80 °C until further analysis.

### Assessment of hepatic steatosis

Hepatic steatosis was assessed in 73 participants from the study group and 21 from the control group using the controlled attenuation parameter (CAP; dB/m), measured with vibration-controlled transient elastography (FibroScan 502 Touch device; Echosens, Paris, France). Examinations were performed in fasting participants in the right liver lobe, and CAP values were expressed as the median of at least 10 valid measurements. Probe selection followed the manufacturer’s recommendations; in adolescents with overweight/obesity, extra large (XL) was used when appropriate to account for increased skin-to-liver capsule distance [20,21].

### Blood biochemical measurements

After an overnight fast, venous blood samples were collected from the cubital vein and analyzed at the Department of Clinical Biochemistry, Children’s Memorial Health Institute (Warsaw, Poland) using a Siemens biochemical analyzer and standard enzymatic methods. Insulin concentrations were determined using a chemiluminescence assay. Homeostatic model assessment of insulin resistance (HOMA-IR) was calculated as follows: HOMA-IR = glucose [mM] × insulin [mIU/l]/22.5.

### 16S rRNA sequencing

Fecal bacterial DNA was isolated using the QIAamp Fast DNA Stool Mini Kit (Qiagen, Hilden, Germany) according to the manufacturer’s protocol, as previously described [22]. Metagenomic libraries targeting the V3–V4 regions of the bacterial 16S rRNA gene were prepared using the Illumina library preparation protocol (Illumina Inc., San Diego, CA, USA). Sequencing was performed on an Illumina MiSeq platform using a 2 × 300-base pair (bp) paired-end run.

### SCFA and AA analysis

SCFAs and AAs were extracted and derivatized as described previously [23], and during sample preparation, calibration standards for SCFAs (formate, acetate, propionate, butyrate, isobutyrate, and valerate) and AAs (alanine, l-arginine, l-cystine, l-glutamic acid, l-leucine, l-lysine, l-serine, l-threonine, l-tyrosine, l-valine, and l-histidine) were added, which allowed the compensation for the effects of the electron spray ionization (ESI) matrix effects, correction, and normalization of the obtained results [24]. Mass spectrometric analyses were performed using an Agilent 7000D Triple Quadrupole system coupled with a 7890 gas chromatography system and a G4513A autosampler (Agilent Technologies, Santa Clara, CA, USA). Separation was achieved using a VF-5ms column (30 m, 0.25 mm, 0.50 μm). Data were acquired in full scan mode [mass/charge ratio (*m*/*z*) 15 to 650] at a frequency of 4.9 scans per second and analyzed using MassHunter software (Agilent Technologies).

### Statistical analysis

Statistical analyses of clinical and biochemical data were performed using GraphPad Prism 11.0.0 (GraphPad Software, Boston, MA, USA). Continuous variables were tested for normality using the Shapiro–Wilk test. Depending on data distribution, group comparisons were performed using Student’s *t* test or Mann–Whitney *U* test. SCFA and AA concentrations were treated as continuous variables and compared between groups using Mann–Whitney *U* test, depending on data distribution. Spearman’s rank correlation analysis was performed between log-transformed AA concentrations and bacterial abundances. Sequencing data were processed using the DADA2 workflow [25] and analyzed in R [25] with additional supporting packages. A compositional data analysis approach was applied, as microbial abundance data are inherently compositional [26,27]. Microbiota β diversity was assessed using centered log-ratio (CLR) transformation and Aitchison distance, and visualized using principal coordinate analysis (PCoA) [26]. Ordination and multivariate analyses were performed using the vegan package [28]. Group comparisons were conducted using analysis of similarities (ANOSIM) and one-factor permutational multivariate analysis of variance (PERMANOVA) with 9,999 permutations [29]. α Diversity was assessed using Chao1, observed richness, Fisher’s alpha, Shannon, and Simpson indices [30–33], and compared using the Wilcoxon rank-sum test. Differential abundance analysis was performed using ANCOM-BC (v. 2.12.0) [34] with Benjamini–Hochberg correction for multiple testing [35]. The pseudo-sensitivity statistic was disabled, and the prevalence cutoff (prv_cut) was set at 20%. Results were compared with those obtained using DESeq2 (v. 1.50.2) [36], with taxa prefiltered to include those present in at least 20% of samples. The differential abundance analyses included only the grouping variable and did not include additional covariates. Data analysis was supported by the following R packages: dada2 [25], phyloseq [37], microbiome [38], vegan [28], ANCOM-BC [34], and DESeq2 [36]. Data visualization was performed using ggplot2 [39] and ggpubr [40].

## Results

### Characteristics of anthropometric and biochemical parameters of adolescents with overweight or obesity

Of 111 participants recruited to this study, 83 had overweight or obesity (the study group) and 28 were normal-weight participants (the control group). Anthropometric parameters of the groups are presented in Table 1. The groups were similar in age and height, and sex was equally distributed. The other parameters differed significantly between the groups (*P* < 0.0001).

**Table 1.** Anthropometric characteristics of the study groups

Characteristic	Control group; *N* = 28 Females = 9 (32.1%); males = 19 (67.9%)		Study group; *N* = 73 Females = 25 (30.1%); males = 58 (69.9%)		*P* test Mann–Whitney
	Median	Range	Median	Range	
Age (years)	15.4	13.3–17.8	15.6	13.2–17.7	0.26335
Height (cm)	174.6	149.8–186.5	172.9	150.3–194.9	0.76748
Height SDS (cm)	0.2	−1.63 to 2.50	0.3	0.77–2.75	0.83593
Weight (kg)	63.1	42.4–77.7	95.0	69.3–137.7	0.00001
Weight SDS	0.3	−1.24 to 1.37	2.3	0.41–4.53	0.00001
BMI (kg/m^2^)	20.4	16.8–23.9	30.5	25.1–52.9	0.00001
BMI SDS	0.0	−1.59 to 1.09	2.6	0.91–3.60	0.00001
Waist circumference (cm)	70.5	58.5–75.0	94.0	76.5–124.5	0.00001
Waist circumference SDS	−0.2	−2.29 to 0.75	2.3	1.30–6.88	0.00001

SDS, standard deviation score

### Blood biochemical parameters

**Fig. 1.**
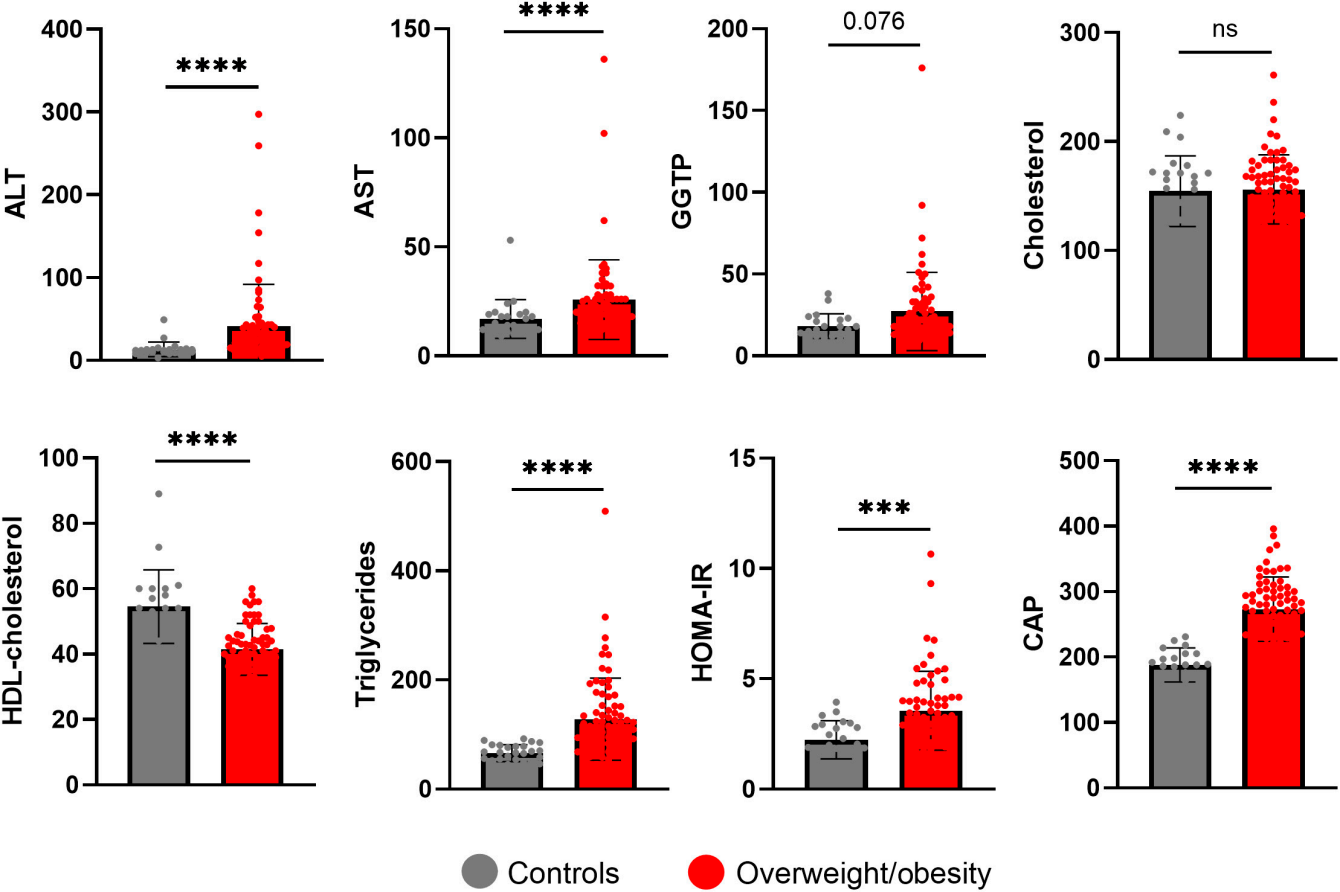
Measures of biochemical parameters and a CAP score in the control and study groups. ALT, alanine aminotransferase; AST, aspartate aminotransferase; GGTP, γ-glutamyl transpeptidase; TG, triglycerides; HDL, high-density lipoprotein; HOMA-IR, homeostatic model assessment of insulin resistance. ns, not significant (*P* ≥ 0.05); ***P < 0.001; ****P < 0.0001.

Significant differences were observed between groups for alanine aminotransferase (ALT), aspartate aminotransferase (AST), triglycerides (TGs), high-density lipoprotein (HDL)-cholesterol, and HOMA-IR. γ-Glutamyl transpeptidase (GGTP) activity showed a trend toward higher values in the study group (*P* = 0.076), whereas total cholesterol levels did not differ significantly between groups (Fig. 1).

### Hepatic steatosis assessment

A noninvasive ultrasound measurement from a FibroScan device that quantifies hepatic steatosis [20] was performed in 73 and 21 participants from the study and control groups, respectively, and the CAP score significantly differentiated adolescents with overweight and obesity from the lean controls (Fig. 1).

#### Bacterial diversity and composition related to overweight and obesity

DNA isolated from all 111 fecal samples was analyzed using 16S rRNA sequencing, but 12 sequencing runs were rejected due to insufficient sequencing quality control. In the remaining 99 sequencing runs, we have mapped an average of 29 059.7 reads per sample. Six [Firmicutes (F), Bacteroidota (B), Proteobacteria, Actinobacteriota, Desulfobacterota, and Verrucomicrobiota] of the 18 identified phyla showed a mean relative abundance greater than 1%. Among the 319 taxa identified (288 of which exceeded 0.01% mean relative abundance), 190 taxa were shared between both groups, whereas 13 were detected exclusively in lean controls and 102 exclusively in overweight or obese subjects. In normal-weight control, the mean B/F ratio in samples was equal to 0.95, and in overweight or obese group, this ratio was 0.97.

**Fig. 2.**
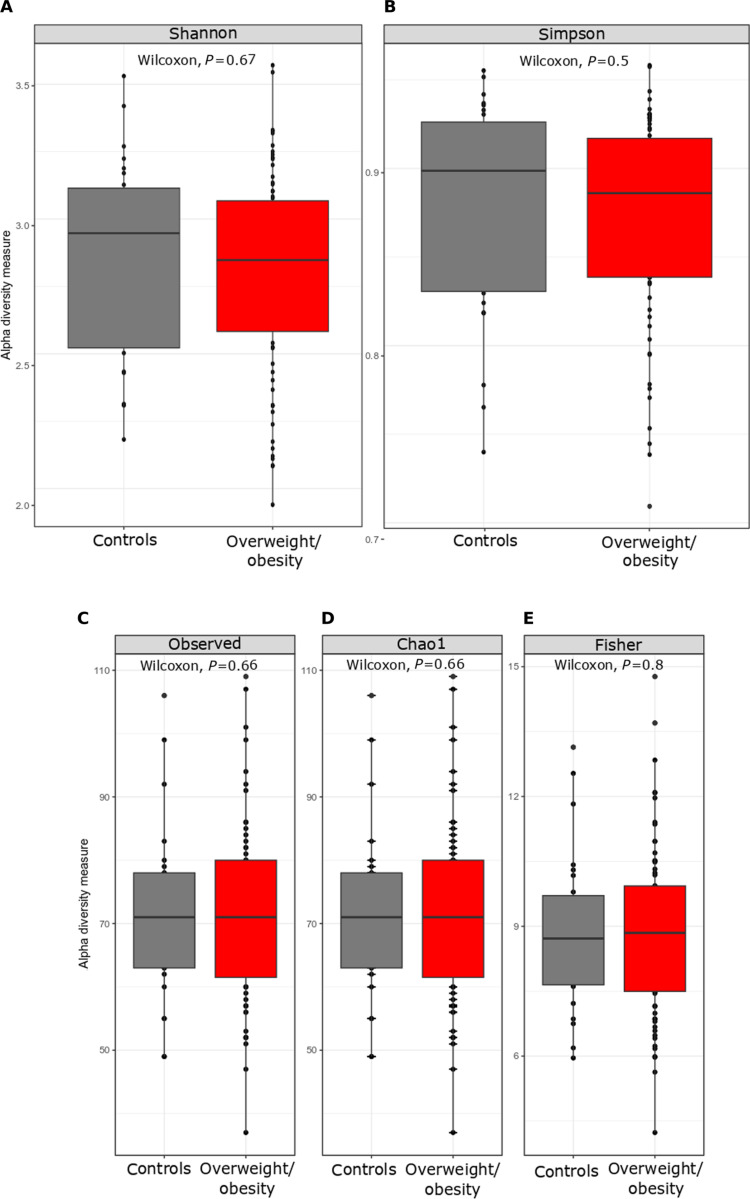
α Diversity analyzed by the Shannon (A), Simpson’s (B), Observed (C), Chao1 (D), and Fisher’s alpha (E) indices in fecal samples collected from individuals with overweight or obesity and lean controls. Data are presented as the mean ± SD. Statistical testing using the Wilcoxon test.

α Diversity was assessed using several indices, including Shannon, Simpson’s, Chao1, Observed alpha, and Fisher’s alpha indices. None of the α-diversity measures showed significant differences in the gut microbiota from overweight or obese subjects compared to the control group (Fig. 2).

**Fig. 3.**
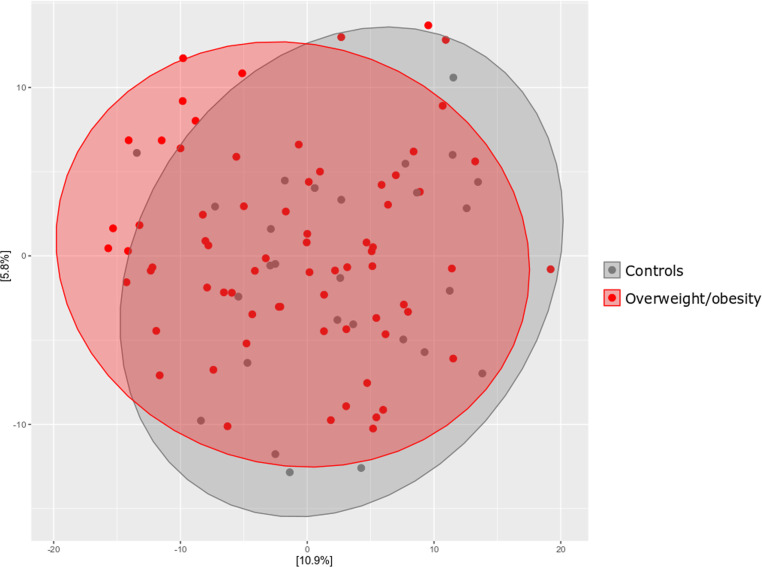
PCoA using with CLR transformation and Aitchison distance of fecal samples collected from individuals with overweight or obesity and lean controls. Each dot represents a single sample, and the number presents the CAP value.

The β diversity of the microbial community, assessed using PCoA, showed a trend toward separation between individuals with overweight or obesity and normal-weight controls (Fig. 3), but neither PERMANOVA test (*P* = 0.80, *R*^2^ statistic = 0.007) nor ANOSIM test. Group differences were evaluated using PERMANOVA and ANOSIM. Neither test detected significant differences between groups (PERMANOVA: *P* = 0.80, *R*^2^ = 0.07; ANOSIM: P = 0.82, *R* statistic = −0.06).

**Fig. 4.**
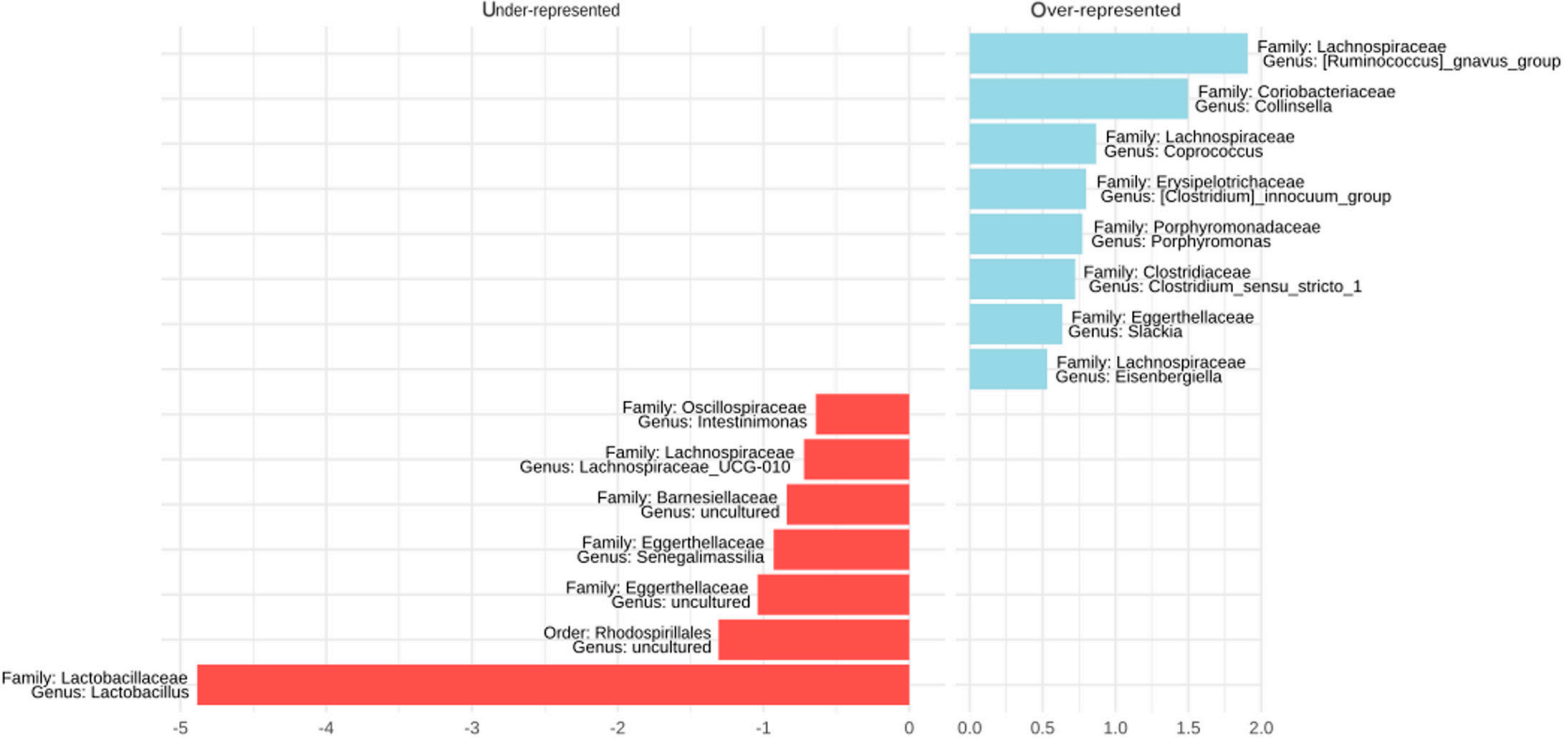
Differentially abundant genera distinguishing adolescents with overweight/obesity from lean controls. Fifteen genera showed significant differences between groups (adjusted *P* < 0.05), with positive log₂ fold-change values indicating over-represented taxa in adolescents with overweight/obesity and negative values indicating under-represented taxa.

Taxonomic analysis of fecal samples identified 15 genera (8 over-represented and 7 under-represented in the overweight/obese group) showing significant differences in abundance (adjusted *P* < 0.05) in overweight or obese subjects compared to lean controls (Fig. 4). The detailed result was attached in Table S1.

#### Metabolite profiles related to overweight and obesity

**Fig. 5.**
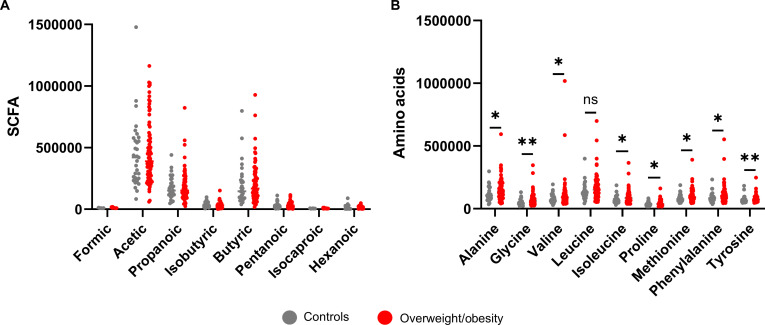
Relative abundance of SCFAs (A) and AAs (B) in fecal extracts of lean controls and participants with overweight or obesity. Statistical significance: ns, not significant (*P* ≥ 0.05); * *P* < 0.05; ** *P* < 0.01.

Gas chromatography–mass spectrometry (GC-MS)-based analyses identified 8 SCFAs—formic acid, acetic acid, propanoic acid, isobutyric acid, butanoic acid, pentanoic acid, and hexanoic acid—and 9 AAs, including alanine, glycine, valine, leucine, isoleucine, proline, methionine, phenylalanine, and tyrosine, in fecal extracts from 79 and 25 participants of the study and control groups, respectively. While the abundances of individual SCFAs did not differ between adolescents with overweight/obesity and lean controls, the relative abundances of all AAs except leucine were significantly higher in the study group compared with controls (Fig. 5).

#### Exploratory correlations between gut microbiota and fecal metabolites in adolescents with overweight and obesity

After FDR correction, no significant correlations were detected between fecal metabolites and bacterial taxa. At the nominal level (*P* < 0.05), 16 moderate correlations were identified in the overweight/obesity group, most of which were negative (rho from −0.39 to −0.12), with a smaller number of positive associations (rho from 0.32 to 0.37). In the control group, several statistically significant correlations were observed, including 9 strong negative correlations, with rho values ranging from −0.59 to −0.50. Additionally, 4 pairs showed moderate negative correlations (rho from −0.45 to −0.43), and 1 pair showed a moderate positive correlation (rho = 0.44). Negative associations were observed for *Porphyromonas* with proline, whereas a moderate positive correlation was detected between *Intestinimonas* and pentanoic acid. In both groups, correlations with the same direction of association were observed for glycine and proline with the *Clostridium innocuum* group. Detailed correlation results are provided in Table S2.

#### Assessment of anthropometric and biochemical parameters related to a CAP score

**Fig. 6.**
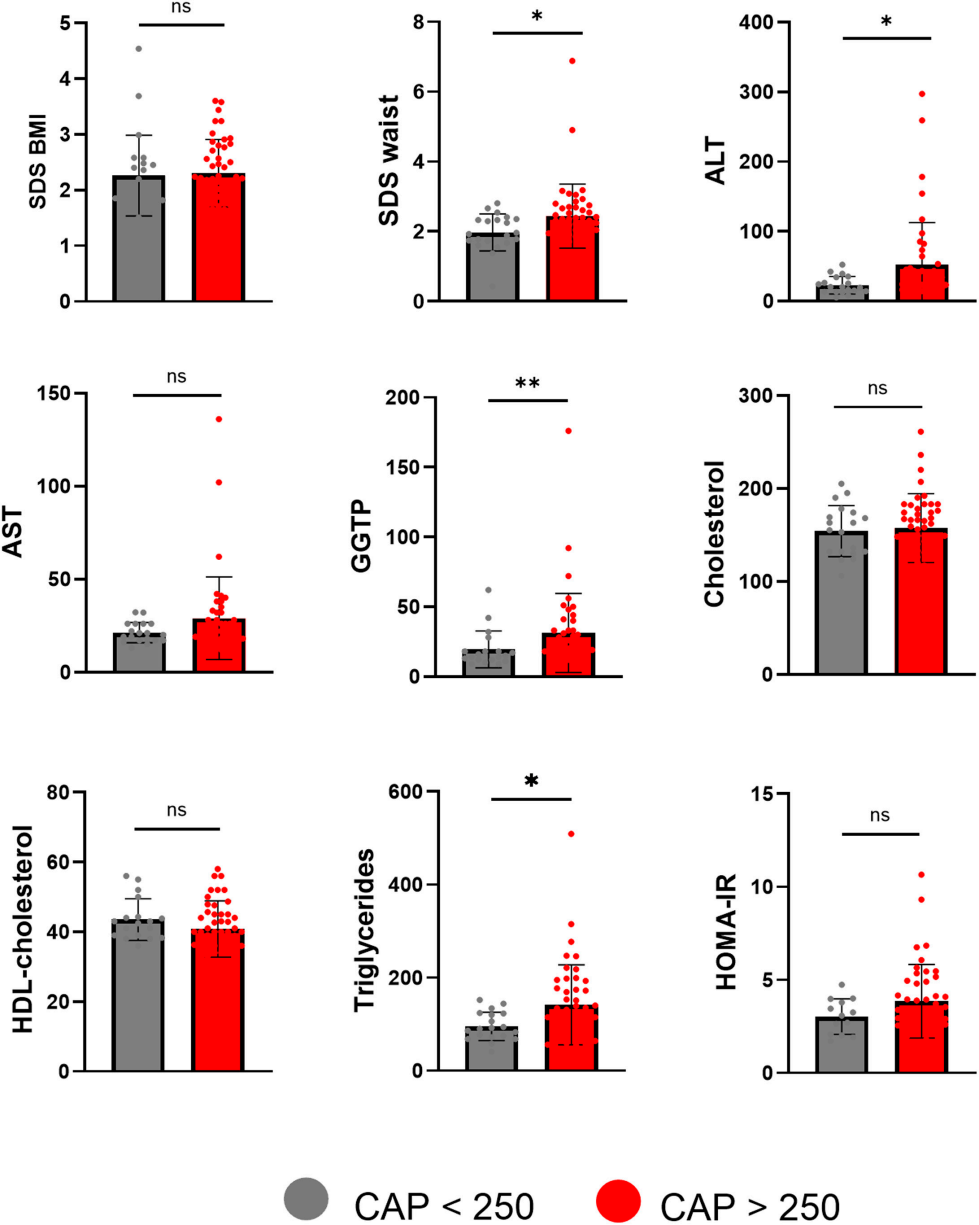
Measures of anthropometric and biochemical parameters in participants with overweight/obesity without fatty liver disease (CAP score ranging from 164 to 242 dB/m) and with fatty liver disease (CAP score ranging from 250 to 396 dB/m). Statistical significance: ns, not significant (*P* ≥ 0.05); * *P* < 0.05; ** *P* < 0.01.

Next, we divided the group of adolescents with overweight or obesity according to the CAP cutoff of 248 dB/m, which aligns with pediatric thresholds for ≥S1 steatosis reported in the literature, although published values remained heterogeneous [20]. For descriptive clarity, this threshold was rounded to 250 dB/m in subgroup presentation. In 22 cases of the study group, the CAP score ranged from 164 to 242 dB/m (median = 222), indicating the absence of fatty liver, whereas in 49 cases, CAP values ranged from 251 to 396 dB/m (median = 292), indicating the presence of fatty liver. Significant differences between these subgroups were observed for waist circumference, ALT, GGTP, and TGs, while body mass index (BMI), AST, total cholesterol, HDL-cholesterol (HDL-C), and HOMA-IR did not differ between cases with overweight or obesity who were categorized by the CAP values (Fig. 6).

#### Bacterial diversity and composition related to fatty liver

Sufficient quality control of the 16S rRNA sequencing runs selected 17 and 41 cases from the study group with the CAP scope lower and higher than 250 dB/m, respectively, and on average, 29 059.7 reads were mapped per sample. Six (Firmicutes, Bacteroidota, Proteobacteria, Actinobacteriota, Desulfobacterota, and Verrucomicrobiota) of the 18 identified phyla showed a mean relative abundance greater than 1%. Among the 319 taxa identified (144 of which exceeded 0.01% mean relative abundance), 192 taxa were shared between both groups, whereas 33 were detected exclusively in lean controls and 55 exclusively in adolescents with overweight or obesity. In obesity group with CAP lower than 250, the mean B/F ratio was equal to 1.01, and in group with CAP higher or equal to 250, this ratio was 0.9 (Wilcoxon *P* value = 0.42).

**Fig. 7.**
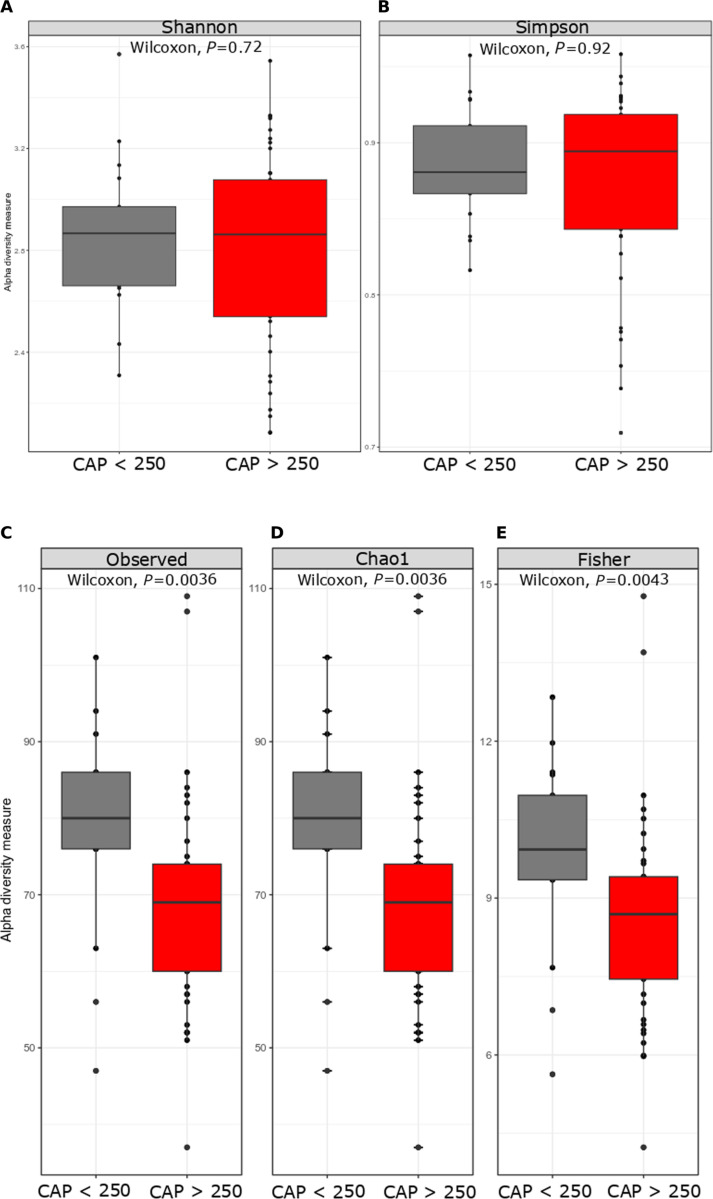
α Diversity analyzed by the Shannon (A), Simpson’s (B), Observed (C), Chao1 (D), and Fisher’s alpha (E) indices in fecal samples collected from individuals with overweight or obesity with and without fatty liver. Data are presented as the mean ± SD. Statistical testing using the Wilcoxon test.

Comparisons of cases from the study group with and without fatty liver showed significant differences in α diversity measured using the Chao1, Observed, and Fisher’s alpha indices between cases with and without fatty liver, whereas the Shannon and Simpson indices did not differ between these subgroups (Fig. 7).

**Fig. 8.**
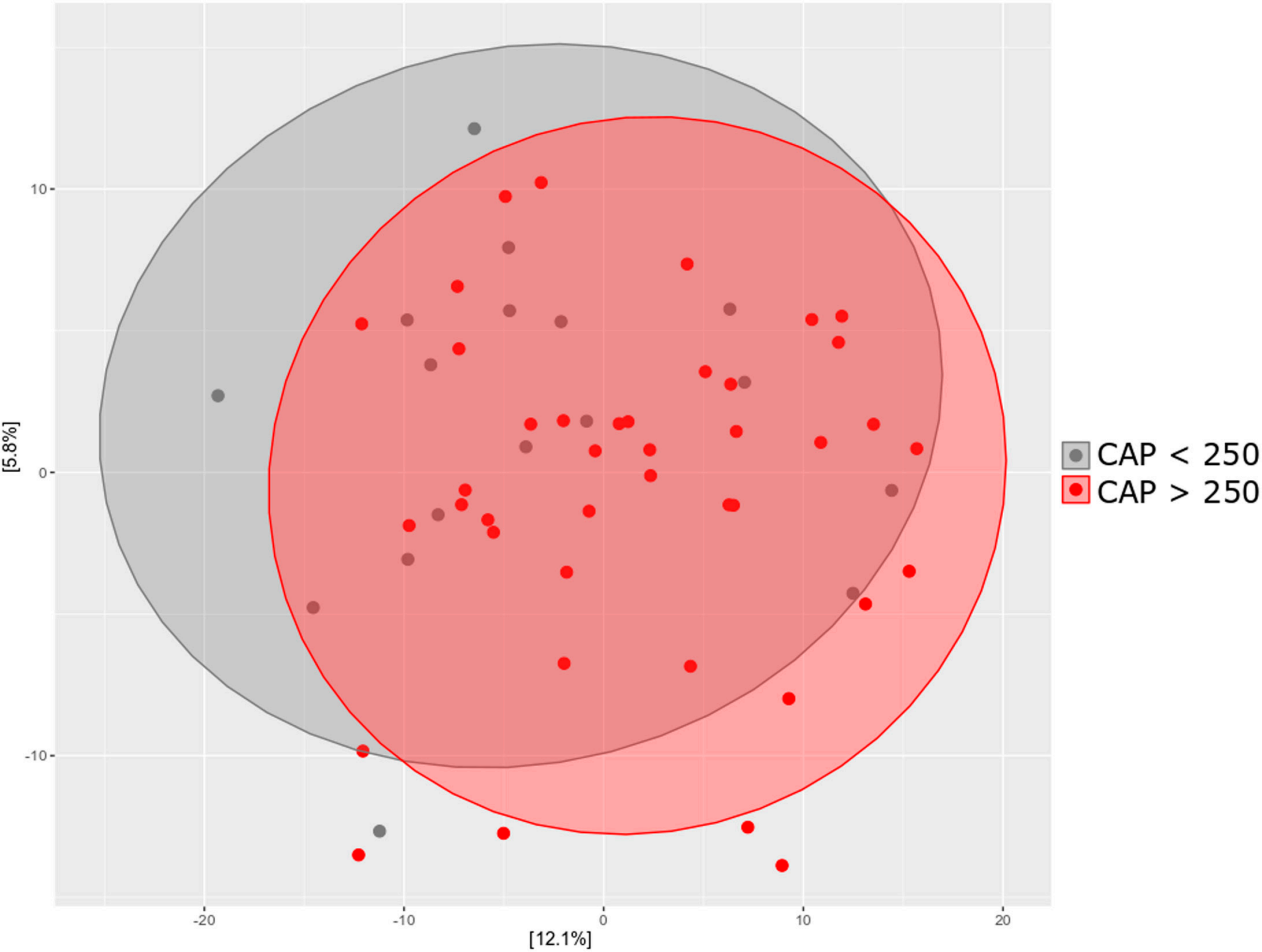
PCoA using with CLR transformation and Aitchison distance of fecal samples collected from individuals with overweight or obesity and lean controls. Each dot represents a single sample, and the label indicates the corresponding CAP value.

**Fig. 9.**
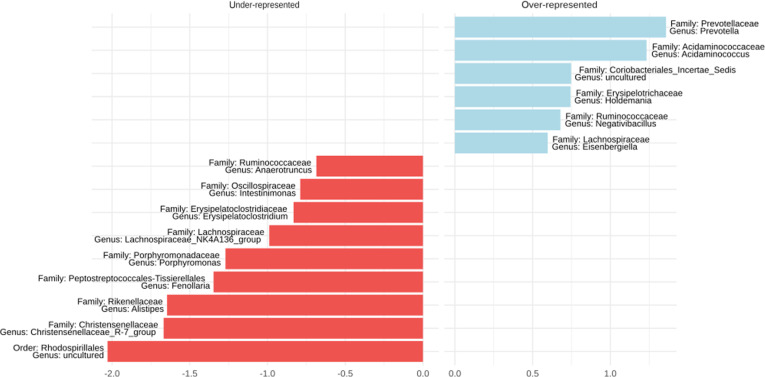
Differentially abundant genera distinguishing adolescents with obesity or overweight with and without fatty liver. Taxonomic analysis identified 15 genera showing significant differences in abundance between subgroups (adjusted *P* < 0.05), with positive log₂ fold-change values indicating taxa over-represented in adolescents with fatty liver and negative values indicating under-represented taxa.

The PERMANOVA test and ANOSIM test did not show significant separation between the cases with and without fatty liver groups (Fig. 8). Finally, taxonomic analysis identified 15 genera (6 over-represented and 9 under-represented in the fatty liver subgroup) showing significant differences in abundance (adjusted *P* < 0.05) between subgroups (Fig. 9). The detailed result was attached in Table S1.

#### Metabolite profiles related to the fatty liver

**Fig. 10.**
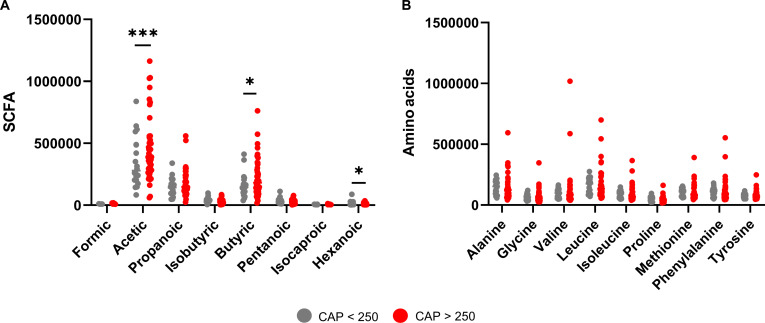
Relative abundance of SCFAs (A) and AAs (B) in fecal extracts of participants with overweight/obesity without fatty liver disease (CAP score ranging from 164 to 242 dB/m) and with fatty liver disease (CAP score ranging from 251 to 396 dB/m). Statistical significance: ns, not significant (*P* ≥ 0.05); * *P* < 0.05; *** *P* < 0.001.

In contrast to significant differences in bacterial diversity and abundance, the relative abundance of AAs did not differ between 20 and 44 adolescents with overweight or obesity, whose CAP scope was lower and higher than 250 dB/m, respectively. Only the relative abundances of acetic acid and butyric acid were significantly higher in the fatty liver subgroup (Fig. 10).

#### Exploratory microbiota–metabolite correlations related to the fatty liver

After FDR correction, no significant correlations were detected between fecal metabolites and bacterial taxa. At the nominal level (*P* < 0.05), multiple correlations were identified in the group with CAP below 250, including 11 strong correlations; for 7 pairs, Spearman’s rho ranged from 0.58 to 0.68, and for the remaining pairs, it was approximately 0.50. Of these, positive correlations were observed between members of *Coriobacteriales* and butyrate and isocaproic acid, whereas negative correlations were detected between *Porphyromonas* and propionate, and between *Prevotella* and butyrate. Similarly, in the group with CAP > 250, we identified 6 moderate correlations. For 3 pairs, rho values ranged from −0.38 to −0.36, and for the other 3 pairs, they ranged from 0.31 to 0.41. Of these, a positive association was noted between *Intestinimonas* and pentanoic acid. Detailed correlation results are provided in Table S2.

## Discussion

Our study contributes to the inconsistent body of evidence on gut microbiota and fecal metabolites in pediatric obesity by providing one of the few analyses conducted in a homogeneous Central European adolescent cohort. We show that despite metabolic disturbances, overall microbial diversity remains largely preserved, while specific low-abundance genera and AAs profiles differentiate individuals with obesity from normal-weight individuals.

MASLD is highly prevalent in both adults and children, affecting one-quarter of the world’s adult population, 3% to 10% of the general pediatric population, and up to 40% of adolescents with obesity [1,41]. Because obesity-related metabolic disturbances are linked to gut dysbiosis, we aimed to compare gut microbiota and fecal metabolites between adolescents with overweight/obesity and those with normal weight, as well as between adolescents with overweight/obesity with and without fatty liver.

We found that, according to the definition, the anthropometric parameters [BMI, BMI standard deviation score (SDS), and waist SDS] as well as results of biochemical testing (serum ALT, AST, TGs, HDL-C, and HOMA-IR) significantly differentiated the overweight/obesity group from the normal-weight control group; none of the 5 α-diversity measures differentiated both groups. Also, both PERMANOVA and ANOSIM tests did not exhibit significant separation of the β diversity between the groups. However, 12 genera differentiated adolescents with overweight/obesity from controls, of which *Lactobacillus*, *Senegalimassilia*, *Lachnospiraceae_UCG-010*, and *Intestinimonas* were underrepresented and *Eisenbergiella*, *Slackia*, *Clostridium_sensu_stricto_1*, *Porphyromonas*, *[Clostridium]_innocuum_group*, *Coprococcus*, *Collinsella*, and *[Ruminococcus]_gnavus_group* were overrepresented in overweight/obese cases. Previous adult and pediatric studies have reported inconsistent findings regarding microbial diversity in obesity, with α diversity described as reduced, increased, or unchanged across cohorts [42]. Similarly, although various taxa including *Streptococcus*, *Acidaminococcus*, *Prevotella*, and *Escherichia Shigella* have been repeatedly associated with obesity, the specific genera differed substantially between studies [42,43]. These discrepancies likely stem from heterogeneous age groups, sequencing platforms, analytical pipelines, and geographical differences, all of which contribute to high variability in reported microbial signatures. This heterogeneity was also typical for our comparisons, underscoring the lack of a universal microbial marker profile of obesity [42,44].

MASLD, formerly known as NAFLD, refers to hepatic steatosis that additionally meets one of 3 criteria: excess adiposity, the presence of prediabetes or type 2 diabetes, or at least 2 metabolic risk symptoms: increased waist circumference, hypertension, hypertriglyceridemia, low HDL-C, impaired fasting glucose, and increased TG to HDL-C ratio [45]. Using the CAP from the FibroScan study, we next divided adolescents with overweight/obesity into those with fatty liver (50 cases) and those without fatty liver (23 cases). Our 2 subgroups differed in waist circumference, ALT, GGT, and TGs, but did not differ in BMI, AST, TGs, HDL-C, and HOMA-IR.

The FibroScan device allows for a noninvasive measurement of fatty liver infiltration and is sufficiently sensitive for clinical use [45–47]. The rationale for specific cutoff points for CAP is based on optimizing the balance between sensitivity and specificity in the diagnosis of fatty liver disease, as graded by liver biopsy (S0 to S3) or magnetic resonance imaging (MRI)–proton density fat fraction [48–54]. Although specific cutoff points may vary between studies, commonly used thresholds for detecting grades of steatosis (S) are as follows: mild (S1) > 248 dB/m, moderate (S2) > 268 dB/m, and severe (S3) > 280 to 290 dB. In practice, a CAP value of <238 dB/m is often considered normal, whereas values >290 dB/m usually suggest severe steatosis [52–54]. However, using FibroScan in adolescents has some limitations. Although studies have demonstrated high sensitivity (over 90%) of CAP in detecting steatosis in children, there is no globally standardized cutoff value for CAP in pediatric populations. In adolescents, adult’s thresholds may be too high, and child-specific cutoffs (approximately 225 to 265 dB/m) have been considered, although standardized guidelines for CAP thresholds in children are still evolving. Differences between adult and adolescent populations may be primarily due to differences in metabolic factors, body composition, and liver pathology [51]. In children and adolescents, CAP is associated with BMI *z* scores and waist circumference *z* scores, not age [51].

α-Diversity measurements using Chao1, Observed, and Fisher’s alpha indices, but not using the Shannon and Simpson indices, showed significant differences between adolescents with overweight/obesity with and without fatty liver disease. Again, we did not observe a significant distinction in α diversity between subgroups, whereas taxonomic analysis revealed an overrepresentation of the genera *Christensenellaceae_R-7_group*, *Erysipelatoclostridium*, *Lachnospiraceae_NK4A136_group*, *Alistipes*, *Fenollaria*, *Porphyromonas*, *Intestinimonas*, and *Anaerotruncus*, and an underrepresentation of *Eisenbergiella*, *Negativibacillus*, *Holdemania*, *Acidaminococcus*, and *Prevotella* in the subgroup with compared to those without fatty liver disease.

Several of the taxa differentiating the study groups have previously been implicated in metabolic dysfunction. *Ruminococcus gnavus*, frequently reported in obesity and inflammatory bowel conditions, has been previously associated with pro-inflammatory potential and mucin degradation [55]. *Collinsella* has been linked to dyslipidemia, insulin resistance, and increased gut permeability in prior studies, which is consistent with its overrepresentation in metabolically altered states [56]. In contrast, members of the *Christensenellaceae* family are typically linked to a “lean” microbial phenotype, making their increased abundance in adolescents with fatty liver an unexpected finding that may reflect age-specific or early-stage metabolic adaptations rather than classical adult-type dysbiosis [57]. Taken together, these observations may support the idea that early metabolic disruption in juveniles is accompanied by subtle compositional changes in low-abundance taxa that may reflect functional differences suggested in previous studies. However, it should be emphasized that the present study does not directly assess functional activity, and therefore, these interpretations remain speculative and are based on associations reported in the literature.

Previous studies of NAFLD in both children and adults have reported highly inconsistent alterations in α diversity, with some showing reduced richness, others increased, and many no change at all [43,58]. Our findings align with this variability, as we did not observe the phylum level shifts described in certain Asian cohorts or the clear reductions in microbial richness frequently reported in pediatric NAFLD [59,60]. The absence of differences in Shannon and Simpson indices suggests that early steatosis changes primarily affect low abundance taxa without disturbing dominant community structure, a pattern consistent with prior pediatric NAFLD studies [58]. Gut-derived metabolites and microbial taxa may influence hepatic lipid accumulation through the gut–liver axis, which is increasingly recognized in pediatric MAFLD.

One of the reasons why studies on obesity and NAFLD fail to identify a consistent microbial signature is the substantial methodological heterogeneity in differential abundance testing [58–60]. In our dataset, ANCOM-BC identified 15 genera differentiating overweight/obese from normal-weight adolescents and another 15 genera distinguishing those with and without hepatic steatosis, whereas DESeq2 detected only 4 and 1 genus, respectively (not shown). Such discrepancies between pipelines have been widely documented; a recent benchmarking study evaluating 14 analytical tools across 38 amplicon datasets reported dramatic variability in the number and identity of important features, with results strongly influenced by sample size, sequencing depth, and effect size [61]. These differences underscore the importance of using compositionality-aware frameworks for microbiome analyses [62]. While the purpose of the present study was to report the outcomes of our statistical comparisons rather than to dissect methodological assumptions, future research would clearly benefit from methodological harmonization. Approaches such as ALDEx2 and ANCOM-II, which consistently perform well across heterogeneous datasets [61], may help improve reproducibility and reduce analytical uncertainty in microbiome studies.

Previous studies [44,63–65] reported elevated levels of AAs, SCFAs, and other metabolites in both overweight and/or obese adults and children. In this study, pairwise comparisons between adolescents with overweight/obesity and normal-weight controls did not reveal statistical differences in the SCFA levels, but found significantly higher levels of all AAs studied, except leucine. Our previously published study [44] reported significant differences for all AAs except leucine and phenylalanine between obese adults and normal-weight controls, of which some were significantly correlated with BMI. The other study performed in obese children found that daily consumption of vegetable and fruit mousses for 24 weeks significantly lowered relative AA abundances [22]. The observation may suggest a shift in colonic metabolic activity toward enhanced protein fermentation rather than alterations in overall fermentative output. Such a pattern has been described in obesity-related dysbiosis, where reduced saccharolytic capacity or changes in substrate availability can increase reliance on proteolytic pathways, potentially leading to the accumulation of AAs and their derivatives in the distal gut [66]. However, because SCFA levels remained comparable between groups, our findings likely indicate a moderate imbalance rather than a profound restructuring of microbial metabolic fluxes. This interpretation is consistent with pediatric studies showing that early obesity is often accompanied by functional microbial alterations that precede major changes in dominant taxa or global fermentative activity. Taken together, these findings may suggest an imbalance in protein- versus carbohydrate-derived fermentation pathways in adolescents with obesity [67].

Dietary proteins and peptides are the major sources of intestinal AAs. Fecal AAs are used by gut bacteria for growth, energy, and the production of signaling molecules (e.g., SCFAs), being crucial for the survival and communication of gut bacteria, in the management of environmental stressors within the gut, and to maintain the gut barrier integrity and immune function [68]. Changes in fecal AA abundances are associated with several human pathologies, including obesity, cancer, inflammatory bowel disease, irritable bowel syndrome, and autism spectrum disorder [69–73]. Glutamine, arginine, and leucine stimulate the mammalian target of rapamycin (mTOR) signaling pathway, alanine acts as a cellular energy sensor via the AMP-activated protein kinase (AMPK), and alanine and arginine modulate the urea cycle, while phenylalanine and tyrosine are linked to the development of metabolic disorders [70]. Obese children exhibit increased fecal concentrations of ammonia and amine, nitrogenous compounds produced by AA deamination and decarboxylation, that are linked with bacterial dysbiosis [74]. As reported previously, an increased abundance in pathways for phenylalanine, tyrosine, tryptophan, and methionine biosynthesis was observed in fecal samples from obese children, while high plasma levels of leucine, isoleucine, and valine were positively correlated with BMI, associating with hepatic lipid accumulation, insulin resistance, and chronic low-grade inflammation in childhood obesity [70,75]. However, elevated fecal AAs may also result from multiple nonmicrobial mechanisms, such as higher dietary protein intake, altered intestinal absorption, accelerated transit time, and host metabolic dysfunction. Since none of these factors have not been investigated in this study, the biological interpretation of the results of our metabolomics studies is definitely incomplete.

The metabolic function of the gut microbiota results mainly from bacterial-related metabolites, of which SCFAs are the largest group generated by the intestinal microbiota. SCFAs serve as energy sources, improve the integrity of the intestinal barrier, and exert anti-inflammatory effects [76,77]. Bacterial metabolic processes in distal parts of the colon may be related to the availability of AAs [78]. While no statistical differences in the levels of AAs were observed between adolescents with overweight/obesity with fatty liver and those without fatty liver, the abundances of acetic acid and butyric acid were found in higher amounts in the fatty liver subgroup. Butyric acid has been reported to provide up to 70% of the energy required by colon epithelial cells, strengthen gut barrier integrity, and exert anti-inflammatory effects [79]. Acetic acid regulates fatty acid and carbohydrate metabolism and generates energy in peripheral tissues after absorbing into the bloodstream; in combination with other SCFAs, it can reduce appetite and influence body weight management and associates with reducing the time for bowel movements [68]. Both acids, along with propionic acid, contribute to lowering the pH of the colon, which inhibits the growth of pathogens and boosts mineral absorption [80]. The interpretation of elevated SCFA levels in pediatric obesity remains complex, as current evidence presents a biologically plausible paradox. On one hand, SCFAs, particularly acetate and butyrate, can enhance energy harvest by increasing substrate availability and promoting hepatic lipogenesis, mechanisms that have been suggested to contribute to excess weight gain in obesity models [81]. On the other hand, these metabolites exert well-established anti-inflammatory and barrier-protective effects, making their overall influence context-dependent rather than uniformly detrimental or beneficial [82]. Therefore, the higher concentrations of acetate and butyrate observed in adolescents with fatty liver in our study may reflect this dual metabolic and immunological role, further highlighting the inconsistent and often contradictory findings reported in pediatric SCFA research. Despite the potential benefits of SCFAs, the overall function of SCFAs in pediatric overweight/obesity is not clear [74]. One of the study reported decreased concentrations of SCFA concentration in obese children [83], while other studies—elevated levels of acetate, propionate, butyrate, and isovalerate [84–86])—positively correlated with BMI *z* scores [86]. While excess SCFAs can be an extra source of energy, involved in developing or maintaining of pediatric overweight/obesity, it can also modulate excretion of insulin via the GPR41/43 pathway [87].

In this study, only weak or moderate correlations were found between gut bacteria and fecal metabolite studied. Impaired interactions between gut microbiome and the related metabolites in childhood obesity may rather result from dysfunction of microbial community than the presence or absence of specific taxa [65]. As discussed previously [44], the human gut microbiome comprises at least 1,800 genera and 15,000 to 36,000 species of bacteria coevolving with the host [88,89], but only a fraction of these bacteria are present in a single individual. In addition, microbiomes of obese individuals show great variability depending also on geographic location [90]. Therefore, creating a clear and universally accepted definition of bacterial dysbiosis is difficult, if at all possible.

The main limitation of this study relates to the general limitations resulting from the lack of a uniform and widely accepted method of analyzing metagenomics studies. Another limitation of this study is the lack of detailed assessment of pubertal stage (e.g., Tanner staging) and sex hormone status. Given that adolescence is a period of dynamic hormonal changes, which may influence gut microbiota composition and metabolic profiles, this factor may have acted as a potential confounder. However, the comparable sex distribution across study groups reduces the likelihood of a major systematic bias. Additionally, the analyses were performed as group-based comparisons and were not adjusted for potential confounders such as age, sex, BMI *z* score, or pubertal stage, which may have influenced the observed associations. Moreover, the imbalance between the number of studied cases and control individuals may have further increased susceptibility to confounding effects, as unequal group sizes can influence statistical robustness [91,92]. This is a cross-sectional study that cannot confirm the causality between the gut dysbiosis and development of hepatic steatosis. Although the sample size is comparable to other studies, larger cohorts might better identify the taxonomic and metabolic profiles. An untargeted shotgun sequencing of the total genomic DNA from an entire microbial community, instead of 16S rRNA sequencing, would better capture the bacterial diversity and microbiome metabolic changes.

Environmental factors and genetic background are fundamental yet distinct factors determining the composition, diversity, and function of the human gut microbiome. While dietary patterns and physical activity are dominant, modifiable factors that can rapidly alter microbial profiles, host genetics play a more subtle, enduring role in shaping the long-term, individualized structure of the microbiome [90,93,94]. Both an obesogenic diet and limited physical activity are believed to contribute to the growing obesity epidemic. On the other hand, although the link between gut microbiota and obesity has gained increasing attention in recent years, the role of gut microbiota changes in the development of obesity has not yet been fully defined [44]. Consequently, the lack of data on dietary habits and lifestyle, which made it difficult to determine the relationship between gut microbiota and metabolites, is another important limitation of this study, which may complicate the interpretation of the reported gut–liver associations.

As mentioned, there are limited research examining the association between gut microbiota changes and simple overweight/obesity or MAFLD in Caucasian children [1]. Our study conducted in a cohort of Polish adolescents with overweight or obesity aimed to fill this gap. Even if presenting with variable overweight/obesity grade, this was a homogeneous population coming from one center limiting influence of external factors. Moreover, children with overweight/obesity, regardless of whether they had fatty liver disease or not, did not differ in terms of BMI waist circumference.

Importantly, pediatric cohorts from Central and Eastern Europe remain markedly underrepresented in microbiome research, particularly in studies addressing obesity and MAFLD. Our analysis provides data from a relatively homogeneous Polish adolescent population, offering insight into a geographic and ethnic group that is rarely included in large pediatric microbiome studies and may display distinct environmental, dietary, and lifestyle influences on gut microbial composition. However, to better verify the role of Western-type diets and lifestyles in the interaction of gut microbiota and metabolites, self-reported diet and physical activity, as well as untargeted metagenomics and metabolomics approaches, would be helpful in elucidating the functional relationships between microbial communities in future studies.

## Conclusion

Our study demonstrates that adolescents with overweight/obesity and those with hepatic steatosis exhibit subtle but potentially biologically relevant alterations in gut microbiota and fecal metabolite profiles, despite largely preserved global microbial diversity. These findings support the notion that early metabolic dysfunction in youth is accompanied primarily by shifts within low-abundance taxa and AA-related metabolic pathways rather than by overt community-level dysbiosis. The distinct signatures identified across obesity- and steatosis-related comparisons may reflect the sensitivity of the adolescent microbiome to early metabolic stress and underscore the potential utility of microbiota-based markers in detecting preclinical stages of metabolic impairment. Future longitudinal and multi-omics studies are needed to clarify causality, identify mechanistic pathways, and determine whether these microbial patterns can serve as targets for personalized prevention or therapeutic strategies in pediatric metabolic health.

## Ethical Approval

The study was approved by the Ethics Committee of the Children’s Memorial Health Institute in Warsaw, Poland (approval no. 34/KBE/2019).

## Data Availability

The datasets presented in this study can be found in online repositories. The data that support the findings of this study were deposited at BioProject repository under accession number PRJNA1450065. The R scripts used for the statistical analyses are available at https://github.com/Addreoran/obesity.

## References

[1] Mierzwa M, Malczyk Ż, Bik-Multanowski M, Brandt-Heunemann S, Flehmig B, Małecka-Tendera E, Mazur A, Petriczko E, Ranke MB, Wabitsch M, et al. High prevalence of metabolic-associated fatty liver disease (MAFLD) in children and adolescents with severe obesity. J Clin Med. 2025;14,(10): 3565. 40429560 10.3390/jcm14103565 PMC12112316

[2] WHO Regional Office for Europe. *WHO European Childhood Obesity Surveillance Initiative (COSI): Report on the sixth round of data collection, 2022–2024*. Copenhagen (Denmark): WHO Regional Office for Europe; 2025.

[3] Obesity and overweight. [accessed 18 Mar 2026] https://www.who.int/news-room/fact-sheets/detail/obesity-and-overweight

[4] Oladipupo SO, Ezenabor EH, Ojo AB, Ogunlakin AD, Ojo OA. Interplay of the pathophysiological mechanisms of non-alcoholic fatty liver disease, diabetes mellitus, and inflammation: A growing threat to public health. Obesity Med. 2025;55: Article 100613.

[5] European Society for Pediatric Gastroenterology, Hepatology and Nutrition (ESPGHAN), European Association for the Study of the Liver (EASL), North American Society for Pediatric Gastroenterology, Hepatology, and Nutrition (NASPGHAN), Latin-American Society for Pediatric Gastroenterology, Hepatology, and Nutrition (LASPGHAN), Asian Pan-Pacific Society for Pediatric Gastroenterology, Hepatology and Nutrition (APPSPGHAN), Pan Arab Society for Pediatric Gastroenterology and Nutrition (PASPGHAN), Commonwealth Association of Paediatric Gastroenterology & Nutrition (CAPGAN), Federation of International Societies of Pediatric Hepatology, Gastroenterology and Nutrition (FISPGHAN). Paediatric steatotic liver disease has unique characteristics: A multisociety statement endorsing the new nomenclature. J Pediatr Gastroenterol Nutr. 2024;78(5):1190–1196. 38529849 10.1002/jpn3.12156

[6] Anderson EL, Howe LD, Jones HE, Higgins JPT, Lawlor DA, Fraser A. The prevalence of non-alcoholic fatty liver disease in children and adolescents: A systematic review and meta-analysis. PLOS ONE. 2015;10(10): Article e0140908. 26512983 10.1371/journal.pone.0140908 PMC4626023

[7] Sood V, Alam S, Nagral A, Srivastava A, Deshmukh A, Bavdekar A, Acharyya BC, Geetha SM, Gupte G, Bhatia I, et al. Practice recommendations for metabolic dysfunction-associated steatotic liver disease by the Indian Society of Pediatric Gastroenterology, Hepatology and Nutrition (ISPGHAN). Indian Pediatr. 2024;61:919–934. 39297398

[8] Omaña-Guzmán I, Rosas-Diaz M, Martínez-López YE, Perez-Navarro LM, Diaz-Badillo A, Alanis A, Bustamante A, Castillo-Ruiz O, Toro-Cisneros ND, Esquivel-Hernandez DA, et al. Strategic interventions in clinical randomized trials for metabolic dysfunction-associated steatotic liver disease (MASLD) and obesity in the pediatric population: A systematic review with meta-analysis and bibliometric analysis. BMC Med. 2024;22:548. 39574069 10.1186/s12916-024-03744-x PMC11580631

[9] Younossi ZM, Zelber-Sagi S, Lazarus JV, Wong VW-S, Yilmaz Y, Duseja A, Eguchi Y, Castera L, Pessoa MG, Oliveira CP, et al. Global consensus recommendations for metabolic dysfunction-associated steatotic liver disease and steatohepatitis. Gastroenterology. 2025;169:1017–1032.e2. 40222485 10.1053/j.gastro.2025.02.044

[10] Younossi ZM, Kalligeros M, Henry L. Epidemiology of metabolic dysfunction-associated steatotic liver disease. Clin Mol Hepatol. 2025;31(Suppl):S32–S50. 39159948 10.3350/cmh.2024.0431 PMC11925440

[11] Boushehri YG, Meymanatabadi Z, Tanha AE, Azami P, Alaei M, Alamdari AA, Momtazi H, Moezzi ND, Habibzadeh A, Khanmohammadi S. Association of triglyceride glucose-body mass index (TyG-BMI) with metabolic dysfunction-associated steatotic liver disease: A systematic review and meta-analysis. PLOS ONE. 2025;20(8): Article e0324483. 40758732 10.1371/journal.pone.0324483 PMC12321072

[12] Jamil A, Chivese T, Elshaikh U, Sendall M. Efficacy of the Mediterranean diet in treating metabolic dysfunction-associated steatotic liver disease (MASLD) in children and adolescents: A systematic review and meta-analysis. BMC Public Health. 2024;24:2701. 39363272 10.1186/s12889-024-19378-w PMC11450996

[13] Jiang L, Du L-D, Zeng J, Chu H-K, Peng Z, Fan J-G. Pediatric metabolic dysfunction-associated steatotic liver disease and the gut microbiome: From research landscape to targeted modulation. Clin Mol Hepatol. 2026;32:53–68. 40825719 10.3350/cmh.2025.0718 PMC12835764

[14] Lin Y-C, Liao F-M, Chao H-C, Chen A-C, Jeng Y-M, Lin C-C, Tiao M-M, Yang Y-J, Yeung C-Y, Chen H-L, et al. Consensus statement on metabolic dysfunction-associated steatotic liver disease in children and adolescents from the joint TASL-TSPGHAN expert committee. JGH Open. 2025;9(6): Article e70137. 40520848 10.1002/jgh3.70137 PMC12162361

[15] Zhang L, Zi L, Kuang T, Wang K, Qiu Z, Wu Z, Liu L, Liu R, Wang P, Wang W. Investigating causal associations among gut microbiota, metabolites, and liver diseases: A Mendelian randomization study. Front Endocrinol. 2023;14:1159148. 10.3389/fendo.2023.1159148 PMC10354516 37476494

[16] Li F, Ye J, Shao C, Zhong B. Compositional alterations of gut microbiota in nonalcoholic fatty liver disease patients: A systematic review and meta-analysis. Lipids Health Dis. 2021;20:22. 33637088 10.1186/s12944-021-01440-w PMC7908766

[17] Lee G, You HJ, Bajaj JS, Joo SK, Yu J, Park S, Kang H, Park JH, Kim JH, Lee DH, et al. Distinct signatures of gut microbiome and metabolites associated with significant fibrosis in non-obese NAFLD. Nat Commun. 2020;11:4982. 33020474 10.1038/s41467-020-18754-5 PMC7536225

[18] Su X, Chen S, Liu J, Feng Y, Han E, Hao X, Liao M, Cai J, Zhang S, Niu J, et al. Composition of gut microbiota and non-alcoholic fatty liver disease: A systematic review and meta-analysis. Obes Rev. 2024;25: Article e13646. 37813400 10.1111/obr.13646

[19] Thallapureddy K, Twitchell D, Ott K, Pedicone LD, Owo C, Kumar N, Gelfond J, Shankar N, Goros M, Kwok D, et al. The accuracy of FibroScan, FIB-4, and nonalcoholic fatty liver disease fibrosis score in predicting biopsy-defined fibrosis and steatosis across all fibrosis stages in patients with metabolic dysfunction associated steatotic liver disease. Medicine. 2025;104: Article e42016. 40295262 10.1097/MD.0000000000042016 PMC12040034

[20] Arteaga I, Chacón C, Martínez-Escudé A, Rojano IR, Diez-Fadrique G, Carmona-Cervelló M, Torán-Monserrat P. Evaluating pediatric NAFLD with controlled attenuation parameter: A comprehensive narrative review. Diagnostics. 2025;15(3):299. 39941229 10.3390/diagnostics15030299 PMC11816681

[21] Sasso M, Beaugrand M, de Ledinghen V, Douvin C, Marcellin P, Poupon R, Sandrin L, Miette V. Controlled attenuation parameter (CAP): A novel VCTE^TM^ guided ultrasonic attenuation measurement for the evaluation of hepatic steatosis: Preliminary study and validation in a cohort of patients with chronic liver disease from various causes. Ultrasound Med Biol. 2010;36(11):1825–1835. 20870345 10.1016/j.ultrasmedbio.2010.07.005

[22] Czarnowski P, Bałabas A, Kułaga Z, Kulecka M, Goryca K, Pyśniak K, Unrug-Bielawska K, Kluska A, Bagińska-Drabiuk K, Głowienka-Stodolak M, et al. Effects of soluble dextrin fiber from potato starch on body weight and associated gut dysbiosis are evident in Western diet-fed mice but not in overweight/obese children. Nutrients. 2024;16(7):917. 38612951 10.3390/nu16070917 PMC11013109

[23] Zeber-Lubecka N, Kulecka M, Balabas A, Czarnowski P, Pyśniak K, Dąbrowska M, Ostrowski J, Hennig EE. Lifetime changes in gut microbiota and metabolite composition in high-fat diet-induced obesity in apolipoprotein A-IV gene knockout mice. Biology. 2025;14, (9): 1278. 10.3390/biology14091278 PMC12467894 41007422

[24] Czarnowski P, Mikula M, Ostrowski J, Żeber-Lubecka N. Gas chromatography–mass spectrometry-based analyses of fecal short-chain fatty acids (SCFAs): A summary review and own experience. Biomedicines. 2024;12(8):1904. 39200366 10.3390/biomedicines12081904 PMC11351285

[25] Callahan BJ, McMurdie PJ, Rosen MJ, Han AW, Johnson AJA, Holmes SP. DADA2: High-resolution sample inference from Illumina amplicon data. *Nat Methods*. 2016;**13**(7):581–583. 10.1038/nmeth.3869 PMC4927377 27214047

[26] Gloor GB, Macklaim JM, Pawlowsky-Glahn V, Egozcue JJ. Microbiome datasets are compositional: And this is not optional. Front Microbiol. 2017;8:2224. 29187837 10.3389/fmicb.2017.02224 PMC5695134

[27] R: The R project for statistical computing. [accessed 18 Mar 2026] https://www.r-project.org/

[28] Oksanen J, Simpson GL, Blanchet FG, Kindt R, Legendre P, Minchin PR, O’Hara B, Solymos P, Stevens MHH, Szoecs E, et al. Vegan: Community ecology package. 2001.

[29] Anderson MJ. A new method for non-parametric multivariate analysis of variance. Austral Ecol. 2001;26:32–46.

[30] Chao A. Nonparametric estimation of the number of classes in a population. Scand J Stat. 1984;11:265–270.

[31] Fisher RA, Corbet AS, Williams CB. The relation between the number of species and the number of individuals in a random sample of an animal population. J Anim Ecol. 1943;12:42.

[32] Shannon CE. A mathematical theory of communication. Bell Syst Tech J. 1948;27(3):379–423.

[33] Simpson EH. Measurement of diversity. Nature. 1949;163:688–688.

[34] Lin H, Peddada SD. Analysis of compositions of microbiomes with bias correction. Nat Commun. 2020;11:3514. 32665548 10.1038/s41467-020-17041-7 PMC7360769

[35] Benjamini Y, Hochberg Y. Controlling the false discovery rate: A practical and powerful approach to multiple testing. J R Stat Soc Ser B. 1995;57:289–300.

[36] Love MI, Huber W, Anders S. Moderated estimation of fold change and dispersion for RNA-seq data with DESeq2. Genome Biol. 2014;15(12):550. 25516281 10.1186/s13059-014-0550-8 PMC4302049

[37] McMurdie PJ, Holmes S. Phyloseq: An R package for reproducible interactive analysis and graphics of microbiome census data. PLOS ONE. 2013;8(4): Article e61217. 23630581 10.1371/journal.pone.0061217 PMC3632530

[38] Introduction to the microbiome R package. [accessed 18 Mar 2026] https://microbiome.github.io/tutorials/

[39] Wickham H. Data analysis. In: Wickham H, editor. *ggplot2: Elegant graphics for data analysis*. Cham: Springer International Publishing; 2016. p. 189–201.

[40] Kassambara A. kassambara/ggpubr. 2026.

[41] Eslam M, George J. Reply to: Correspondence on “A new definition for metabolic associated fatty liver disease: An international expert consensus statement”: MAFLD: Moving from a concept to practice. J Hepatol. 2020;73(5):1268–1269. 32800588 10.1016/j.jhep.2020.06.036

[42] Pinart M, Dötsch A, Schlicht K, Laudes M, Bouwman J, Forslund SK, Pischon T, Nimptsch K. Gut microbiome composition in obese and non-obese persons: A systematic review and meta-analysis. Nutrients. 2021;14:12. 35010887 10.3390/nu14010012 PMC8746372

[43] Klimenko ES, Belkova NL, Rychkova LV, Darenskaya MA, Tugarinova OA, Semenova NV, Savinova YS, Bugun OV, Balzhirova DB, Kolesnikova LI. Alpha diversity indices as indicators of the variability of gut microbiota in obese adolescents of different ethnicities. Bull Exp Biol Med. 2024;176:591–594. 38724810 10.1007/s10517-024-06073-4

[44] Kulecka M, Jaworski P, Zeber-Lubecka N, Bałabas A, Piątkowska M, Czarnowski P, Frączek B, Tarnowski W, Mikula M, Ostrowski J. The gut microbiome obesity index: A new analytical tool in the metagenomics workflow for the evaluation of gut dysbiosis in obese humans. Nutrients. 2025;17(14):2320. 40732945 10.3390/nu17142320 PMC12298630

[45] Eslam M, Alkhouri N, Vajro P, Baumann U, Weiss R, Socha P, Marcus C, Lee WS, Kelly D, Porta G, et al. Defining paediatric metabolic (dysfunction)-associated fatty liver disease: An international expert consensus statement. Lancet Gastroenterol Hepatol. 2021;6:864–873. 34364544 10.1016/S2468-1253(21)00183-7

[46] Goyal P, Chopra R, Goyal O. Do hepatic fibrosis and steatosis measured by hepatic transient elastography (FibroScan) predict cardiovascular risk in patients with non-alcoholic fatty liver disease: An observational cross-sectional study. Indian J Cardiovasc Dis Women. 2024;9:66–73.

[47] Xu X, Jin J, Liu Y. Performance of FibroScan in grading steatosis and fibrosis in patients with nonalcoholic fatty liver disease: A meta-analysis. Arab J Gastroenterol. 2023;24:189–197. 37996351 10.1016/j.ajg.2023.08.003

[48] Ahn SB, Jun DW, Kang B-K, Kim M, Chang M, Nam E. Optimal cutoff value for assessing changes in intrahepatic fat amount by using the controlled attenuation parameter in a longitudinal setting. Medicine. 2018;97(50): Article e13636. 30558054 10.1097/MD.0000000000013636 PMC6320035

[49] Cao Y, Xiang L, Qi F, Zhang Y, Chen Y, Zhou X. Accuracy of controlled attenuation parameter (CAP) and liver stiffness measurement (LSM) for assessing steatosis and fibrosis in non-alcoholic fatty liver disease: A systematic review and meta-analysis. EClinicalMedicine. 2022;51: Article 101547. 35844772 10.1016/j.eclinm.2022.101547 PMC9284399

[50] Pu K, Wang Y, Bai S, Wei H, Zhou Y, Fan J, Qiao L. Diagnostic accuracy of controlled attenuation parameter (CAP) as a non-invasive test for steatosis in suspected non-alcoholic fatty liver disease: A systematic review and meta-analysis. BMC Gastroenterol. 2019;19:51. 30961539 10.1186/s12876-019-0961-9 PMC6454693

[51] Sirli R, Sporea I. Controlled attenuation parameter for quantification of steatosis: Which cut-offs to use? Can J Gastroenterol Hepatol. 2021;2021:6662760. 33834008 10.1155/2021/6662760 PMC8018863

[52] Nastasa R, Stanciu C, Zenovia S, Singeap A-M, Cojocariu C, Sfarti C, Girleanu I, Chiriac S, Cuciureanu T, Huiban L, et al. The prevalence of liver steatosis and fibrosis assessed by vibration-controlled transient elastography and controlled attenuation parameter in apparently healthy Romanian medical students. Diagnostics. 2021;11(12):2341. 34943578 10.3390/diagnostics11122341 PMC8700151

[53] Taru M-G, Neamti L, Taru V, Procopciuc LM, Procopet B, Lupsor-Platon M. How to identify advanced fibrosis in adult patients with non-alcoholic fatty liver disease (NAFLD) and non-alcoholic steatohepatitis (NASH) using ultrasound elastography—a review of the literature and proposed multistep approach. Diagnostics. 2023;13(4):788. 36832276 10.3390/diagnostics13040788 PMC9955630

[54] Arteaga I, Chacón C, Martínez-Escudé A, Ruiz Rojano I, Diez Fadrique GS, Carmona Cervelló M, Torán-Monserrat P. Evaluating pediatric NAFLD with controlled attenuation parameter: A comprehensive narrative review. Diagnostics. 2025;15(3):229. 39941229 10.3390/diagnostics15030299 PMC11816681

[55] Crost EH, Coletto E, Bell A, Juge N. Ruminococcus gnavus: Friend or foe for human health. FEMS Microbiol Rev. 2023;47:fuad014. 37015876 10.1093/femsre/fuad014 PMC10112845

[56] Gomez-Arango LF, Barrett HL, Wilkinson SA, Callaway LK, McIntyre HD, Morrison M, Nitert MD. Low dietary fiber intake increases Collinsella abundance in the gut microbiota of overweight and obese pregnant women. Gut Microbes. 2018;9:189–201. 29144833 10.1080/19490976.2017.1406584 PMC6219589

[57] Tavella T, Rampelli S, Guidarelli G, Bazzocchi A, Gasperini C, Pujos-Guillot E, Comte B, Barone M, Biagi E, Candela M, et al. Elevated gut microbiome abundance of *Christensenellaceae, Porphyromonadaceae and Rikenellaceae* is associated with reduced visceral adipose tissue and healthier metabolic profile in Italian elderly. Gut Microbes. 2021;13:1–19. 10.1080/19490976.2021.1880221 PMC7889099 33557667

[58] Tokuhara D. Role of the gut microbiota in regulating non-alcoholic fatty liver disease in children and adolescents. Front Nutr. 2021;8:700058. 34250000 10.3389/fnut.2021.700058 PMC8267179

[59] Tsai M-C, Liu Y-Y, Lin C-C, Wang C-C, Wu Y-J, Yong C-C, Chen K-D, Chuah S-K, Yao C-C, Huang P-Y, et al. Gut microbiota dysbiosis in patients with biopsy-proven nonalcoholic fatty liver disease: A cross-sectional study in Taiwan. Nutrients. 2020;12(3):820. 32204538 10.3390/nu12030820 PMC7146257

[60] Yun Y, Kim H-N, Lee E-J, Ryu S, Chang Y, Shin H, Kim H-L, Kim T-H, Yoo K, Kim HY. Fecal and blood microbiota profiles and presence of nonalcoholic fatty liver disease in obese versus lean subjects. PLOS ONE. 2019;14(3): Article e0213692. 30870486 10.1371/journal.pone.0213692 PMC6417675

[61] Nearing JT, Douglas GM, Hayes MG, MacDonald J, Desai DK, Allward N, Jones CM, Wright RJ, Dhanani AS, Comeau AM, et al. Microbiome differential abundance methods produce different results across 38 datasets. Nat Commun. 2022;13(1):342. 35039521 10.1038/s41467-022-28034-z PMC8763921

[62] Calgaro M, Romualdi C, Waldron L, Risso D, Vitulo N. Assessment of statistical methods from single cell, bulk RNA-seq, and metagenomics applied to microbiome data. Genome Biol. 2020;21(1):191. 32746888 10.1186/s13059-020-02104-1 PMC7398076

[63] Jaimes JD, Slavíčková A, Hurych J, Cinek O, Nichols B, Vodolánová L, Černý K, Havlík J. Stool metabolome-microbiota evaluation among children and adolescents with obesity, overweight, and normal-weight using 1H NMR and 16S rRNA gene profiling. PLOS ONE. 2021;16(3): Article e0247378. 33765008 10.1371/journal.pone.0247378 PMC7993802

[64] Cui M, Trimigno A, Castro-Mejía JL, Reitelseder S, Bülow J, Bechshøft RL, Nielsen DS, Holm L, Engelsen SB, Khakimov B. Human fecal metabolome reflects differences in body mass index, physical fitness, and blood lipoproteins in healthy older adults. Meta. 2021;11:717. 10.3390/metabo11110717 PMC8620003 34822375

[65] Therdtatha P, Gruneck L, Nachalam P, Jinatham V, Saninjuk K, Nakayama J, Popluechai S. Gut microbiome and fecal metabolite profiles in obese school-aged children from northern Thailand. Front Microbiol. 2025;16:1657839. 41019529 10.3389/fmicb.2025.1657839 PMC12461226

[66] Patra D, Banerjee D, Ramprasad P, Roy S, Pal D, Dasgupta S. Recent insights of obesity-induced gut and adipose tissue dysbiosis in type 2 diabetes. Front Mol Biosci. 2023;10:1224982. 37842639 10.3389/fmolb.2023.1224982 PMC10575740

[67] Chen X, Sun H, Jiang F, Shen Y, Li X, Hu X, Shen X, Wei P. Alteration of the gut microbiota associated with childhood obesity by 16S rRNA gene sequencing. PeerJ. 2020;8: Article e8317. 31976177 10.7717/peerj.8317 PMC6968493

[68] Lange O, Proczko-Stepaniak M, Mika A. Short-chain fatty acids—A product of the microbiome and its participation in two-way communication on the microbiome-host mammal line. Curr Obes Rep. 2023;12(2):108–126. 37208544 10.1007/s13679-023-00503-6 PMC10250490

[69] Jagt JZ, Struys EA, Ayada I, Bakkali A, Jansen EEW, Claesen J, van Limbergen JE, Benninga MA, de Boer NKH, de Meij TGJ. Fecal amino acid analysis in newly diagnosed pediatric inflammatory bowel disease: A multicenter case-control study. Inflamm Bowel Dis. 2022;28:755–763. 34757415 10.1093/ibd/izab256 PMC9074868

[70] Zamosteanu D, Filip N, Trandafir LM, Ţarcă E, Pertea M, Bordeianu G, Bernic J, Heredea AM, Cojocaru E. Current data on the role of amino acids in the management of obesity in children and adolescents. Int J Mol Sci. 2025;26(15):7129. 40806262 10.3390/ijms26157129 PMC12346589

[71] Opperman RCM, Vermeer E, Bosch S, de Meij TGJ, de Boer NKH, Struys EA. Faecal amino acids stability: Investigating optimal sampling conditions for analysis. Metabolomics. 2025;21(4):81. 40515879 10.1007/s11306-025-02279-3 PMC12167247

[72] Chamtouri M, Merghni A, Salazar N, Redruello B, Gaddour N, Mastouri M, Arboleya S, de los Reyes-Gavilán CG. An overview on fecal profiles of amino acids and related amino-derived compounds in children with autism spectrum disorder in Tunisia. Molecules. 2023;28(7):3269. 37050030 10.3390/molecules28073269 PMC10096484

[73] Kulecka M, Czarnowski P, Bałabas A, Turkot M, Kruczkowska-Tarantowicz K, Żeber-Lubecka N, Dąbrowska M, Paszkiewicz-Kozik E, Walewski J, Ługowska I, et al. Microbial and metabolic gut profiling across seven malignancies identifies fecal *Faecalibacillus intestinalis* and formic acid as commonly altered in cancer patients. Int J Mol Sci. 2024;25(15):8026. 39125593 10.3390/ijms25158026 PMC11311272

[74] Zhang S, Dang Y. Roles of gut microbiota and metabolites in overweight and obesity of children. Front Endocrinol. 2022;13:994930. 10.3389/fendo.2022.994930 PMC9492854 36157438

[75] Yu D, Richardson NE, Green CL, Spicer AB, Murphy ME, Flores V, Jang C, Kasza I, Nikodemova M, Wakai MH, et al. The adverse metabolic effects of branched-chain amino acids are mediated by isoleucine and valine. Cell Metab. 2021;33(5):905–922.e6. 33887198 10.1016/j.cmet.2021.03.025 PMC8102360

[76] Oliphant K, Allen-Vercoe E. Macronutrient metabolism by the human gut microbiome: Major fermentation by-products and their impact on host health. Microbiome. 2019;7:91. 31196177 10.1186/s40168-019-0704-8 PMC6567490

[77] Frampton J, Murphy KG, Frost G, Chambers ES. Short-chain fatty acids as potential regulators of skeletal muscle metabolism and function. Nat Metab. 2020;2(9):840–848. 32694821 10.1038/s42255-020-0188-7

[78] Macfarlane S, Quigley ME, Hopkins MJ, Newton DF, Macfarlane GT. Polysaccharide degradation by human intestinal bacteria during growth under multi-substrate limiting conditions in a three-stage continuous culture system. FEMS Microbiol Ecol. 1998;26:231–243.

[79] Hodgkinson K, El Abbar F, Dobranowski P, Manoogian J, Butcher J, Figeys D, Mack D, Stintzi A. Butyrate’s role in human health and the current progress towards its clinical application to treat gastrointestinal disease. Clin Nutr. 2023;42:61–75. 36502573 10.1016/j.clnu.2022.10.024

[80] Bushell FML, Tonner PD, Jabbari S, Schmid AK, Lund PA. Synergistic impacts of organic acids and pH on growth of *Pseudomonas aeruginosa*: A comparison of parametric and Bayesian non-parametric methods to model growth. Front Microbiol. 2019;9:3196. 30671033 10.3389/fmicb.2018.03196 PMC6331447

[81] May KS, den Hartigh LJ. Gut microbial-derived short chain fatty acids: Impact on adipose tissue physiology. Nutrients. 2023;15(2):272. 36678142 10.3390/nu15020272 PMC9865590

[82] Garzon-Escamilla N, Medina-Cardena M, Roy P, Trent J, Jamous J, Somesan Y, Denslow SJ. Mechanistic links between the gut microbiome and longevity therapeutics. Biomedicine. 2026;14:316. 10.3390/biomedicines14020316 PMC12937842 41751214

[83] Barczyńska R, Litwin M, Sliżewska K, Szalecki M, Berdowska A, Bandurska K, Libudzisz Z, Kapuśniak J. Bacterial microbiota and fatty acids in the faeces of overweight and obese children. Pol J Microbiol. 2018;67(3):339–345. 30451451 10.21307/pjm-2018-041 PMC7256813

[84] Payne AN, Chassard C, Zimmermann M, Müller P, Stinca S, Lacroix C. The metabolic activity of gut microbiota in obese children is increased compared with normal-weight children and exhibits more exhaustive substrate utilization. Nutr Diabetes. 2011;1(7):e12. 23154580 10.1038/nutd.2011.8 PMC3302137

[85] Gyarmati P, Song Y, Dotimas J, Yoshiba G, Christison A. Cross-sectional comparisons of gut microbiome and short-chain fatty acid levels among children with varied weight classifications. Pediatr Obes. 2021;16(6): Article e12750. 33174684 10.1111/ijpo.12750

[86] Riva A, Borgo F, Lassandro C, Verduci E, Morace G, Borghi E, Berry D. Pediatric obesity is associated with an altered gut microbiota and discordant shifts in Firmicutes populations. Environ Microbiol. 2017;19:95–105. 27450202 10.1111/1462-2920.13463 PMC5516186

[87] Murugesan S, Nirmalkar K, Hoyo-Vadillo C, García-Espitia M, Ramírez-Sánchez D, García-Mena J. Gut microbiome production of short-chain fatty acids and obesity in children. Eur J Clin Microbiol Infect Dis. 2018;37(4):621–625. 29196878 10.1007/s10096-017-3143-0

[88] Sender R, Fuchs S, Milo R. Revised estimates for the number of human and bacteria cells in the body. PLOS Biol. 2016;14(8): Article e1002533. 27541692 10.1371/journal.pbio.1002533 PMC4991899

[89] Mikó E, Kovács T, Sebő É, Tóth J, Csonka T, Ujlaki G, Sipos A, Szabó J, Méhes G, Bai P. Microbiome-microbial metabolome-cancer cell interactions in breast cancer-familiar, but unexplored. Cells. 2019;8:293. 30934972 10.3390/cells8040293 PMC6523810

[90] Medina DA, Li T, Thomson P, Artacho A, Pérez-Brocal V, Moya A. Cross-regional view of functional and taxonomic microbiota composition in obesity and post-obesity treatment shows country specific microbial contribution. Front Microbiol. 2019;10:2346. 31681211 10.3389/fmicb.2019.02346 PMC6812679

[91] Oldfield M. Unequal sample sizes and the use of larger control groups pertaining to power of a study. *Dstl*. 2016;1.

[92] Alamolhoda M, Ayatollahi SMT, Bagheri Z. A comparative study of the impacts of unbalanced sample sizes on the four synthesized methods of meta-analytic structural equation modeling. BMC Res Notes. 2017;10(1):446. 28877742 10.1186/s13104-017-2768-5 PMC5585956

[93] Wang B, Kong Q, Li X, Zhao J, Zhang H, Chen W, Wang G. A high-fat diet increases gut microbiota biodiversity and energy expenditure due to nutrient difference. Nutrients. 2020;12(10):3197. 33092019 10.3390/nu12103197 PMC7589760

[94] Huda MN, Salvador AC, Barrington WT, Gacasan CA, D’Souza EM, Deus Ramirez L, Threadgill DW, Bennett BJ. Gut microbiota and host genetics modulate the effect of diverse diet patterns on metabolic health. Front Nutr. 2022;9: Article 896348. 36061898 10.3389/fnut.2022.896348 PMC9434023

